# Increased variability of motor cortical excitability to transcranial magnetic stimulation in migraine: a new clue to an old enigma

**DOI:** 10.1007/s10194-011-0379-4

**Published:** 2011-09-01

**Authors:** Adriana B. Conforto, Mariana S. Moraes, Edson Amaro, William B. Young, Lais A. Lois, André L. Gonçalves, Mario F. P. Peres

**Affiliations:** 1 Instituto Israelita de Ensino e Pesquisa, Hospital Israelita Albert Einstein, Av. Albert Einstein, 627/2SS, Morumbi, São Paulo, SP 05652-900 Brazil; 2 Hospital das Clínicas da Faculdade de Medicina da Universidade de São Paulo, São Paulo, Brazil; 3 Jefferson Headache Center, Department of Neurology, Thomas Jefferson University Hospital, Philadelphia, USA

**Keywords:** Migraine, Magnetic stimulation, Neurophysiology, Motor physiology, Physiology

## Abstract

Increased, decreased or normal excitability to transcranial magnetic stimulation (TMS) has been reported in the motor (M1) and visual cortices of patients with migraine. Light deprivation (LD) has been reported to modulate M1 excitability in control subjects (CS). Still, effects of LD on M1 excitability compared to exposure to environmental light exposure (EL) had not been previously described in patients with migraine (MP). To further our knowledge about differences between CS and MP, regarding M1 excitability and effects of LD on M1 excitability, we opted for a novel approach by extending measurement conditions. We measured motor thresholds (MTs) to TMS, short-interval intracortical inhibition, and ratios between motor-evoked potential amplitudes and supramaximal M responses in MP and CS on two different days, before and after LD or EL. Motor thresholds significantly increased in MP in LD and EL sessions, and remained stable in CS. There were no significant between-group differences in other measures of TMS. Short-term variation of MTs was greater in MP compared to CS. Fluctuation in excitability over hours or days in MP is an issue that, until now, has been relatively neglected. The results presented here will help to reconcile conflicting observations.

**Electronic supplementary material:**

The online version of this article (doi:10.1007/s10194-011-0379-4) contains supplementary material, which is available to authorized users.

## Introduction

Abnormal cortical excitability is an intriguing piece in the puzzle of migraine pathogenesis. While strong data support increase in activity or excitability in the cerebral cortex measured in visual and motor areas of patients with migraine (MP), there is also evidence that decreased excitability leading to decreased preactivation and lack of habituation to afferent stimuli is migraine’s biological signature [1–4]. Inconsistencies between these mixed results have not yet been resolved.

A widely used, powerful, and non-invasive tool to evaluate cortical excitability in humans is transcranial magnetic stimulation (TMS) (for a review, see [5]). In MP, visual and corticospinal or cortico-cortical excitabilities to TMS have been found to be increased, decreased, or normal, compared to control subjects without migraine (CS) [1–4, 6, 7]. The debate was therefore not settled by these studies probably because, in all of them, only single measures of excitability were performed in MP and CS. Interestingly, variability of visual cortical excitability measured once a day on different days is greater in MP than in CS [7, 8]. This finding raises the important point that increased fluctuation, rather than mere increase or decrease in excitability, may be a marker of abnormal neuronal function in migraine. However, whether this phenomenon is restricted to the visual cortex in MP, and whether it occurs within a day or over several days, is unknown.

In contrast to the conflicting evidence regarding cortical excitability to TMS, a well-recognized feature in MP is the abnormal responsiveness to external stimuli such as environmental light [9]. It is known that light deprivation (LD) modulates visual [10, 11] and motor cortex (M1) [12] excitability to TMS. Effects of LD on M1 excitability in MP have not been described.

This is the first study to compare variability of motor cortex excitability within a day and across several days in MP and CS, before and after LD or exposure to environmental light exposure (EL). We hypothesized that: (1) variability of excitability would be greater in MP compared to CS; (2) LD would increase excitability to a greater extent in MP than in CS.

## Methods

### Subjects

Twenty-six women participated in the study: 17 MP (mean age ± standard error (SE), 35.2 ± 2.8 years), 9 CS (34.1 ± 4.1 years). Inclusion criteria for MP were: diagnosis of migraine (with aura, without aura, or chronic) according to the International Headache Society criteria [13], and at least one migraine attack in the month before the experiments.

Exclusion criteria for all subjects were: left-handedness according to the Oldfield inventory [14]; abnormal brain magnetic resonance imaging; contraindications to TMS [15]; psychiatric conditions other than anxiety or depression; neurological conditions; in the last 4 weeks, use of prophylactic migraine drugs (beta-blockers, calcium channel blockers, antidepressants, or antiepileptic drugs), or any drugs known to interfere in excitability to TMS [16].

Potential CS were excluded if they had a history of any headache during lifetime that fulfilled criteria for a migraine attack according to ICH criteria, any primary headache other than episodic tension-type headache, or any headache in the month before the experiments. Migraine is more common in women than in men, so it was not surprising that all MP volunteers were women [17]. To avoid differences in gender composition between the groups, male gender was an exclusion criteria in the CS group. The experimental protocol was approved by the ethics committee and conformed to ethical standards described in the Declaration of Helsinki. All subjects provided written informed consent.

In MP, migraine history averaged (±SE) 21.1 ± 2.5 years and the mean number (±SE) of days with pain per month was 13.8 ± 1.6. Median MIDAS (Migraine Disability Assessment Score) [18] was IV (range I–IV) and median usual pain intensity (analog score 0–10) was 8 (range 6–10). Ten MP had episodic and seven had chronic migraine (Table 1). Patient 15 fulfilled IHS criteria for chronic migraine but had fewer than 15 days of pain in the month prior to TMS experiments (partial remission).

**Table 1:** Measures of excitability to transcranial magnetic stimulation in patients with migraine

MP	Type of migraine	MT Pre-EL	MT Post-EL	MT Pre-LD	MT Post-LD	SICI Pre-EL	SICI Post-EL	SICI Pre-LD	SICI Post-LD	*N* Pre-EL	*N* Post-EL	*N* Pre-LD	*N* Post-LD
1	Episodic^a^	45	50	44	46	49.3	29.9	42.6	74.3	18	21	16	14
2	Episodic^a^	36	38	35	34	110.9	61.1	36.1	49.4	21	21	19	19
3	Episodic^a^	28	27	35	34	57.5	53.5	29.6	33.9	25	25	17	19
4	Episodic^b^	35	41	31	32	11.4	27.2	47.2	21.6	52	31	21	16
5	Episodic^b^	39	40	42	44	21.6	43.1	59.9	66.1	19	15	64	19
6	Episodic^b^	36	36	35	36	117.8	209.2	105.0	88.2	15	24	25	21
7	Episodic^b^	45	50	44	46	147.3	135.3	182.5	141.6	18	25	15	30
8	Episodic^b^	37	38	40	40	24.0	70.1	20.9	35.7	25	20	17	18
9	Episodic^b^	63	61	58	58	69.7	65.5	91.7	104.9	23	17	21	23
10	Episodic^b^	39	41	39	46	9.2	8.3	38.1	8.1	18	25	27	15
11	Chronic	28	31	29	31	72.7	76.09	37.3	21.1	24	15	23	17
12	Chronic	50	50	49	49	28.9	17.7	33.4	33.6	14	13	17	24
13	Chronic	75	75	68	72	31.9	107.4	51.6	51.1	40	22	21	18
14	Chronic	30	33	32	33	57.6	112.2	31.9	129.4	25	25	18	20
15	Chronic	50	55	42	55	10.5	9.5	10.5	2.9	18	20	25	25
16	Chronic	34	37	36	35	99.9	134.3	82.0	86.5	23	14	16	19
17	Chronic	34	33	35	35	11.3	37.3	33.2	92.9	25	20	22	21
	Mean	41.4	43.3	40.8	42.7	54.8	70.5	54.9	61.3	23.7	20.8	22.6	19.9
	S.E.	2.9	2.8	2.3	2.5	9.8	12.4	9.4	9.5	2.1	1.1	2.6	0.9

Motor thresholds (MT, % of stimulator’s output), short-interval intracortical inhibition (SICI, %), and number of stimuli (*N*) in determination of MT in patients with migraine pre- and post-standard room light exposure (EL) and pre- and post-light deprivation (LD)

^a^With aura

^b^Without aura

*SE* standard error

### Experimental design

**Fig. 1:**
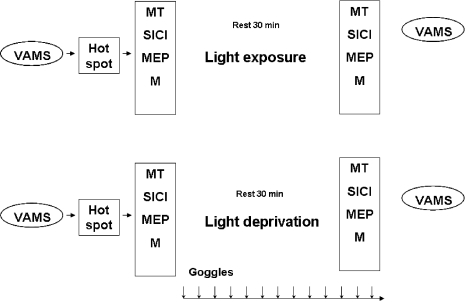
Experimental paradigm. Visual Analog Mood Scores (VAMS) and measures of excitability were performed before (*left*) and after (*right*) environmental light exposure (EL, *top*) and light deprivation (LD, *bottom*) sessions in patients with migraine (MP) and in control subjects (CS). In the EL session, subjects were exposed to ambient light. In the LD session, goggles were kept in place for 30 min and until measurements of excitability were finished. After finding the hot spot to register motor-evoked potentials (MEP) in the right abductor pollicis brevis muscle, motor threshold (MT), short-interval intracortical inhibition (SICI), MEP amplitudes at intensities of 0.9, 1.1, and 1.3 MT, and amplitudes of supraxamaximal M responses (M) were evaluated before and after EL or LD. In both sessions, subjects were instructed to remain at rest with eyes open

Every subject participated in two TMS sessions in a crossover experimental design (Fig. 1). In all experiments, subjects were set at rest, comfortably seated. Transcranial magnetic stimulation was performed before and after 30 min of either LD or EL. This LD or EL extent was chosen because it was reported that 30 min of LD caused an increase in motor cortex excitability in CS [12].

During LD, after the initial TMS measurements under standard room lighting conditions (530 lux), ambient luminance was reduced to a level just sufficient for the investigator to perform TMS; subjects wore opaque goggles and reported complete absence of perceived light. In EL sessions, subjects were only exposed to standard room lighting conditions (530 lux). Therefore, LD was the specific intervention and EL, the control condition. In both sessions, subjects listened to standard songs during the 30 min of LD or EL and were instructed to remain awake during TMS measurements with their eyes open [12, 19]. These instructions were important to avoid sleepiness as the subjects remained with eyes closed, in a relatively resting condition for the 30-min period of LD or EL experiments.

The order of the sessions was randomized across subjects. The maximum interval between experiments was 1 month and all experiments were performed between 1 p.m. and 6 p.m. There were no significant differences between the CS and MP groups regarding the intervals between the two TMS sessions (*p* = 0.87): 6.8 ± 1.7 days in MP and 6.3 ± 2.2 days in CS. In the MP group, there were no significant differences (*p* = 0.73) in the number of days between the last migraine attack and the LD (2.9 ± 0 days) or EL (3.0 ± 1.1 days) sessions. One patient did not recall the exact date of her last migraine episode before LD and another one before EL. Patient 16 reported mild, dull headache, different from migraine, during EL. The pain subsided spontaneously. Patients 7 and 13 reported headache in both experimental sessions, patient 9 in the EL session and patient 12 in the LD session. None of the patients reported auras during or up to 24 h before the experiments.

### TMS

Transcranial magnetic stimulation biphasic pulses were delivered to the “hot spot” of the left M1 for the right *abductor pollicis brevis* (APB) through a MC-B70 figure-of-eight coil (2 × 100 mm, 31kT/s) connected to a MagPro X100 magnetic stimulator (Alpine Biomedical). The coil was placed tangentially to the scalp, with the intersection of both wings at a 45° angle with the midline. Electromyographic (EMG) activity was recorded from surface electrodes placed over the APB muscle, the responses amplified (1,000), filtered (2 Hz–2 kHz), and recorded on a computerized data acquisition system built with the LabVIEW graphical programming language (sampling rate 5 kHz). Its conditional triggering feature was used to deliver TMS stimuli only when the APB muscle was relaxed (EMG activity at baseline <50 μV peak-to-peak amplitude for at least 1 s) [20].

After identification of the APB hot spot, the following TMS measurements [5] were obtained at baseline and immediately after LD and EL sessions: (a) Resting motor threshold (MT), a measure of corticomotor excitability defined as the minimum TMS intensity (measured to the nearest 1% of the maximum output of the magnetic stimulator and delivered randomly 5–7 s apart) required to elicit at least three out of six motor-evoked potentials (MEP) ≥50 μV in consecutive trials, as previously reported [21, 22]. Transcranial magnetic stimulation stimulus intensities were expressed relative to the MT measured from the APB. Numbers of stimuli to determine MT were recorded for each subject in each condition. (b) Short-interval intracortical inhibition (SICI) was measured with paired-pulse TMS. SICI likely reflects intracortical function in GABAergic inhibitory interneurons [5]. Conditioning stimulus intensity was set to 80% of the APB MT. The intensity of the test stimulus was that required to evoke MEPs of approximately 0.5–1 mV (MEP_TS_). This procedure was described by Kujirai in the classical paired-pulse paradigm [23]. The order of presentation of inhibitory (2 ms) and control trial (test stimulus alone) intervals was randomized across subjects. Twelve paired and 12 control trials were recorded. Results were expressed as average percentages of MEP amplitudes in conditioning trials and in test trials (MEP_conditioning stimuli + test stimuli_/MEP _test stimuli_,%). (c) MEP peak-to-peak amplitudes at intensities of 0.9, 1.1, and 1.3 MT. The order of stimulus intensities was randomized across subjects. Results are expressed relative to the maximal peripheral M response peak-to-peak amplitudes (MEP/M, %). M responses were obtained by supramaximal stimulation of the median nerve at the wrist. MEP amplitudes were expressed relative to the amplitude of the maximal peripheral M response. This measurement allows controlling for differences in muscle bulk and electrode position across subjects and reflects the extent of activation of the spinal motor neuron pool of a target muscle, by a single TMS pulse at a given stimulus intensity. Ten trials were performed for each stimulation intensity. One CS refused M response recording and the results from one MP were excluded due to technical problems (MEPs were not saved).

All experiments were performed at the same phase of the menstrual cycle, or in active dosage and withdrawal phases in each subject taking low dosage oral contraceptives, because previous studies showed that, even though MT are unchanged by hormonal levels in both MP and CS, there are differences in SICI measured in the follicular phase compared to the luteal phase in CS [24, 25].

### Subjective states

To evaluate modulation of subjective states by the experimental interventions, Visual Analog Mood Scale (VAMS) of Norris translated into Portuguese [26] was evaluated before and after the LD and EL experimental sessions. VAMS consists of 16 analog scale items printed in a single page. Each item is composed of a pair of opposite adjective words, with a horizontal 100-mm line in between the words. For each item, subjects were requested to mark a point on the line with the distance to each word proportional to his/her feelings at that moment. Cluster analysis grouped the items into four factors: (1) cognitive impairment, composed of the items quick-witted/mentally slow, proficient/incompetent, energetic/lethargic, clear-headed/muzzy, gregarious/withdrawn, well-coordinated/clumsy, and strong/feeble; (2) anxiety, made of the items calm/excited, relaxed/tense, and tranquil/troubled; (3) sedation, composed of alert/drowsy and attentive/dreamy; (4) discomfort, made of interested/bored, happy/sad, contented/discontented, and amicable/antagonistic.

### Statistical analysis

Data are presented as mean ± SE if normally distributed and as median (range) otherwise. Ages of CS and MP were compared with unpaired *t* tests. Intervals before the last migraine episode in LD and EL sessions were compared using Wilcoxon tests.

Motor threshold, short-interval intracortical inhibition, number of stimuli for MT determination, and VAMS scores were analyzed with repeated-measures analysis of variance (ANOVA_RM_) using the factors GROUP (CS and MP), TIME (pre and post), CONDITION (LD and EL), and ORDER OF SESSION (first session, LD; or first session, EL). The factor “ORDER” was included in the model to evaluate carryover bias in the crossover design used in this study.

MEP/M ratios were analyzed with repeated-measures analysis of variance with factors GROUP, TIME, CONDITION, STIMULUS INTENSITY (0.9, 1.1, and 1.3rMT), and ORDER OF SESSION.

Tukey’s post hoc tests with adjusted *p* values were performed when appropriate. *p* values <0.05 were considered to be statistically significant. SAS 9.1 and SPSS 17.0 were used for statistical analysis.

## Results

### Measures of cortical excitability

#### Motor thresholds

Motor threshold results and the number of stimuli given to each subject are shown in Tables 1 and 2. ANOVA_RM_ revealed significant interactions GROUP × TIME [*F*_(1,24)_ = 6.28; *p* = 0.02] and GROUP × ORDER [*F*_(1,24)_ = 5.43; *p* = 0.03]. There were no significant effects of CONDITION or interactions between other factors (*p* > 0.05).

**Table 2:** Measures of excitability to transcranial magnetic stimulation in control subjects

CS	MT Pre-EL	MT Post-EL	MT Pre-LD	MT Post-LD	SICI Pre-EL	SICI Post-EL	SICI Pre-LD	SICI Post-LD	*N* Pre-EL	*N* Pre-EL	*N* Pre-LD	*N* Post-LD
1	33	32	29	31	40.1	23.8	55.1	44.5	44	26	19	17
2	36	34	33	36	54.9	28.0	51.1	36.2	17	41	24	16
3	56	51	58	58	23.8	7.5	15.0	5.3	22	19	13	13
4	57	58	56	56	67.1	38.0	14.9	36.5	20	43	50	18
5	39	40	41	43	145.9	113.9	86.5	86.6	24	18	14	20
6	34	34	31	35	38.3	28.3	63.9	52.7	21	21	21	51
7	45	42	51	43	106.8	46.55	194.2	120.8	29	14	16	17
8	30	30	30	28	69.5	65.8	47.2	40.5	20	17	20	25
9	31	31	31	31	33.8	32.9	41.0	46.1	32	31	17	17
Mean	40.1	39.1	40.0	40.1	64.5	42.8	63.2	52.1	25.4	25.6	21.6	21.6
SE	3.2	3.1	3.7	3.4	12.4	9.8	17.0	10.4	2.6	3.3	3.5	3.6

Motor thresholds (MT, % of stimulator’s output), short-interval intracortical inhibition (SICI, %), and number of stimuli (*N*) in determination of MT in control subjects pre- and post-standard room light exposure (EL) and pre- and post-light deprivation (LD)

*SE* standard error

**Fig. 2:**
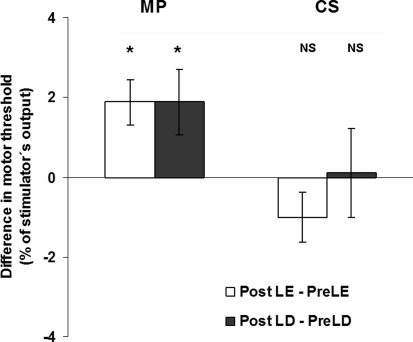
Changes in motor thresholds. Absolute differences between motor thresholds (in percentage of stimulator’s output) achieved for the light exposure (EL) session (*Post-EL*–*Pre-EL*, *white columns*) and for light deprivation (LD) session (*Post-LD*–*Pre-LD*, *black columns*) in patients with migraine (MP) and control subjects (CS). *p < 0.05. *NS* nonsignificant

Post hoc analysis showed that although MTs did not change significantly in CS (*t* = 0.46; *p* = 0.97), they increased significantly in MP after either LD or EL when compared with results obtained before these interventions (*t* = −3.72; *p* = 0.0061) (Fig. 2). There were no significant effects of ORDER revealed by post hoc Tukey’s tests (*p* = 0.085 for CS and *p* = 0.964 for MP).

Next, we evaluated whether the number of stimuli for MT determination was comparable in the LD and EL sessions. No significant effects of GROUP, TIME, CONDITION, ORDER, or interactions between these factors (*p* > 0.05) were found.

These results demonstrate that MTs were less stable in MP than in CS over a short period of time, and that LD or EL conditions did not affect this measurement in both groups, regardless of whether LD or EL was the first experimental session to be performed.

#### Short-interval intracortical inhibition

Short-interval intracortical inhibition results are shown in Tables 1 and 2. ANOVA_RM_ revealed significant interactions GROUP × CONDITION × ORDER [*F*_(1,24)_ = 5.41; *p* = 0.03)] and GROUP × TIME [(*F*_(1,24)_ = 6.23; *p* = 0.021)], while post hoc analysis did not show any significant effects (*p* > 0.05 for all comparisons).

#### MEP/M ratios

There were no significant effects of GROUP, TIME, or ORDER in MEP/M ratios, or interactions between any of these factors (*p* > 0.05). However, a significant effect was found for STIMULUS INTENSITY [*F*_(2,44)_ = 134.6; *p* ≤ 0.001]. As expected, MEP/M ratios increased at greater stimulus intensities in both groups (*p* < 0.001 for all comparisons). In addition, there was a significant effect of CONDITION [*F*_(1,23)_ = 5.75; *p* = 0.026]. Overall, MEP/M ratios were significantly higher in the EL session compared to the LD session (*t* = 2.08; *p* = 0.049).

These results are shown in Supplementary Table 1.

### Subjective states

Supplementary Tables 2 and 3 show VAMS results.

ANOVA_RM_ revealed significant effects of TIME [*F*_(1,24)_ = 4.48; *p* = 0.046] and CONDITION [*F*_(1,24)_ = 4.46; *p* = 0.046] regarding VAMS scores for cognitive impairment. In both groups, scores increased after either LD or EL sessions and were lower in EL compared to LD sessions.

ANOVA_RM_ revealed a significant interaction CONDITION × TIME [*F*_(1,24)_ = 6.3; *p* = 0.019] regarding anxiety scores. There were no significant effects of GROUP, ORDER, or other interactions between any of the factors analyzed (*p* > 0.05). Differences in anxiety scores were not significant according to post hoc analysis: pre-EL × post-EL, *p* = 0.196; pre-LD × post-LD, *p* = 0.136; pre-LD × pre-EL, *p* = 0.354; post-LD × post-EL, *p* = 0.078.

There were no significant effects or interactions regarding VAMS scores for sedation or discomfort (*p* > 0.05).

In summary, cognitive impairment was greater at the end of the experimental sessions in both groups, although it was more prominent in the LD than in the EL sessions. There were no measurable changes in anxiety, sedation, or discomfort between groups, across time or conditions.

## Discussion

The main result of this study was that MT increased significantly within a short period of time (less than 2 h) in MP, but remained stable in CS. This change cannot be attributed to differences in muscle relaxation, because the computer-controlled system used to trigger the magnetic stimulator insured that TMS pulses were administered only at rest, during all MT measurements. Furthermore, there were no significant between-group differences regarding cognitive impairment, anxiety, sedation, or discomfort. The lack of significant differences in MT in the CS group reported here is in agreement with previous reports involving measurements repeated seven times within 10 h [27] and once daily on three different days [27–29], indicating the stability of MT in CS.

Repetitive testing was crucial to observe this MT variability and may explain discrepant results in previous reports [1–4]. It is reasonable to think that if MT is less stable in MP than in CS, differences between groups may or may not arise depending on when the punctual measurements are collected. Our results indicate that controversies about excitability to TMS in MP may partially be due to this factor.

Baseline MTs, measured before LD and EL on different days did not significantly differ in MP in our study, in contrast to phosphene thresholds that were shown to vary significantly more in MP than CS across days [7, 8]. One plausible explanation is that fluctuation in visual cortex excitability does not necessarily parallel fluctuation in M1 excitability. Phosphene thresholds measured once a day were shown to increase, 1–2 days before migraine attacks in children, while MT measured once a day did not significantly change in the interictal period [6]. Resting MT and phosphene thresholds were found not to correlate significantly in healthy subjects in a number of studies [30–33] even though a significant correlation was found between active MT (measured during voluntary muscle contraction) and phosphene thresholds when similar thresholding procedures were employed for both measurements of excitability [34].

Also, phosphene threshold is known to be more variable than MT across and within subjects [7, 8, 33] and depends on subjective responses, while MT relies on objective determination of MEPs. Finally, the use of different types of stimulators and coils limit result comparability between our results and those of studies that evaluated phosphene thresholds in MP and CS.

The magnitude of change in MT in the MP group was small (mean around 2%) but significant. MT is a fairly stable TMS measure that can change significantly after brain lesions, such as stroke or amyotrophic lateral sclerosis [35, 36], or administration of drugs that interfere on ionic channels or on NMDA receptors [16]. On average, MT increases in 2–10% after administration of antagonists of sodium and calcium channels [16, 37–41] and decreases in 2.7–6.7% after administration of the NMDA antagonist ketamine [42]. Therefore, the magnitude of change in MT even after administration of these drugs is relatively small. Considering that no overt structural lesions and only subtle changes in brain excitability would be expected in migraine, we obviously did not anticipate large shifts in MT in MP.

The significant increase in MT observed over a short period of time in MP may reflect abnormal function of ionic channels [1, 3], because MT can be significantly increased by antagonists of sodium and calcium channels [16] and for this reason is considered a marker of ion channel excitability in the motor cortex. Furthermore, mutations of neuronal ionic channels have been identified in rare, familial forms of migraine [43–45]. Neurons with these mutations can be hypoexcitable and hyperexcitable at different points in time, i.e., their excitability is more variable than in non-mutated neurons [46]. Hence, increased variability in neuronal excitability due to abnormal function of ionic channels is a candidate explanation to our findings of greater drifts in MT in MP, compared to CS.

Motor thresholds depend not only on the activity of ion channels of motor cortical neurons [16, 27], but is also influenced by other factors: corticospinal fiber orientation, distance between the coil and the motor cortex, technique of measurement (coil positioning, type of coil or magnetic stimulator), excitability of spinal motor neurons, and possibly by attention, hormonal fluctuations, and fatigue. None of these other factors explain our findings.

First, within-subject comparisons were performed in MT measurements and, therefore, corticospinal fiber orientation was constant. Second, stimulation technique, including the number of stimuli for MT determination, was comparable in all TMS sessions. Although we did not use neuronavigation, it is unlikely that such an approach would have provided a different explanation because a previous report demonstrated no significant differences in MT measured with or without neuronavigation [47]. Third, all experiments were performed in the afternoon and in the same phase of the menstrual cycle in each participant, and subjective states were comparable between the two MP and CS groups. Fourth, no between-group differences were found in M responses (data not shown), arguing against spinal mechanisms.

In contrast with MT results, there were no significant differences between groups with regard to SICI or MEP/M ratios. MEP/M ratios were significantly lower under a condition of less cognitive demand (rest in the dark in the LD session), consistent with previous reports [19, 48]. A limitation to interpretation of these findings is the extreme heterogeneity in SICI and MEP/M ratios within and across subjects. This was not unexpected given the reports about variability of these measures in CS [10, 19, 49].

Motor thresholds were reported to remain unchanged while MEP amplitudes were reported to increase and SICI to decrease after 30 min of LD compared to baseline EL measured in a different experimental session in CS [12]. In contrast, LD had no significant effects on SICI or on MEP/M ratios in CS or in MP in our study. The reason behind this discrepancy between studies is likely the difference in experimental designs: measurements were performed once in each session in the study of Leon-Sarmiento et al. [12], at baseline in the EL session and after 30 min of LD in the LD session. In our study, CS and MP remained at rest for 30 min during exposure to environmental light in the EL session, and without exposure to light in the LD session. Measurements were performed before and after LD and EL. Rest influences baseline activity in the brain. The magnitude of the “rest” condition may have exceeded effects of LD on MT, therefore obscuring any possible effects of this intervention compared to EL.

Another question that arises from our research is whether there is a correlation between the degree of fluctuation in excitability and clinical features (number, duration, severity of attacks, use of prophylactic drugs, gender, and pain during TMS). Most studies on single measurements of MT excluded data from patients having migraine attacks at intervals ranging from 1 week to 24 h before and/or after experiments. This was based on pseudo-normalization of neurophysiological measures performed with various techniques other than TMS [1–4]. However, there is no demonstration that results of these tests mirror excitability measured with TMS. Furthermore, the hypothesis that migraine attacks influence MT failed to be confirmed in children [6] and has not been formally tested in adults. Future studies should include greater sample sizes, provide detailed information of migraine attacks for prolonged periods, and perform more than one MT measurement, within a day and across days.

One exciting, possible application of TMS in a paroxysmal disorder such as migraine is to define surrogate end points for responsiveness to specific therapeutic interventions. In patients with epilepsy, seizure control after 1 year of treatment with antiepileptic drugs can be predicted from early increase in MT and intracortical inhibition measured with TMS after several weeks of treatment [50]. It is possible that change or rate of change in MT may be useful markers to predict responsiveness of MP to pharmacological or non-pharmacological interventions. Moreover, TMS itself has been suggested to be a potential novel non-pharmacological intervention to treat MP, due to beneficial effects reported after single-pulse stimulation of the visual cortex in patients with migraine with aura [51, 52]. If TMS is to be used to predict clinical improvements, then MT and fluctuation in MT are candidate measurements. More studies are necessary to define whether TMS can be an adjuvant tool to stratify patients for specific therapeutic strategies.

Although our study encompassed patients with migraine without aura, with aura, and chronic migraine, we were able to find significant differences between M1 excitability in MP, compared to CS. Whether variability in MT differs in different types of migraine or according to severity of this condition is a matter to be addressed in future studies. In addition, whether it predisposes to migraine attacks or is a consequence of them remains a difficult question.

The results presented here show for the first time that fluctuation in MT is greater in MP compared to CS. Fluctuation in excitability over hours or days in MP is an issue that, until now, has been relatively neglected and is important to understanding contradictory findings of previous studies that performed single measures of excitability. Many reasons may underlie the stability of electric activity in cortical neurons, such as the types, quantity, and activity of ionic channels and the relative strengths of inhibitory and excitatory synaptic inputs [53]. Adding fluctuation in cortical excitability to the complex equation of brain electrical dynamics in migraine will reconcile conflicting results, which may be useful to enlighten the pathogenesis underlying this condition.

## Electronic supplementary material

Below is the link to the electronic supplementary material.

Supplementary material 1 (DOC 103 kb)

Supplementary material 2 (DOC 77 kb)

Supplementary material 3 (DOC 82 kb)
