# Supplementary material for: Increased variability of motor cortical excitability to transcranial magnetic stimulation in migraine: a new clue to an old enigma
Source: J Headache Pain. 2011 Sep 1;13(1):29–37. doi: 10.1007/s10194-011-0379-4 (PMC3253159; doi:10.1007/s10194-011-0379-4)
Supplement: Supplementary file 1 — Supplementary material 1 (DOC 103 kb) [file 10194_2011_379_MOESM1_ESM.doc]

| **MP** |  | **0.9 rMT** | |  |  | **1.1 rMT** | |  | **1.3 rMT** | | | |
| --- | --- | --- | --- | --- | --- | --- | --- | --- | --- | --- | --- | --- |
|  | **Pre EL** | **Post**  **EL** | **Pre**  **LD** | **Post LD** | **Pre**  **EL** | **Post EL** | **Pre**  **LD** | **Post**  **LD** | **Pre**  **EL** | **Post**  **EL** | **Pre**  **LD** | **Post**  **LD** |
| **1** | 0.62 | 0.54 | 0.21 | 0.54 | 1.19 | 7.65 | 2.65 | 11.55 | 12.28 | 30.36 | 15.43 | 27.38 |
| **2** | 0.23 | 0.41 | 0.28 | 0.20 | 1.90 | 6.02 | 0.81 | 0.46 | 5.25 | 7.10 | 4.47 | 3.73 |
| **4** | 0.13 | 0.26 | 0.18 | 0.15 | 0.13 | 2.39 | 0.31 | 0.77 | 2.28 | 4.29 | 4.05 | 6.96 |
| **5** | 0.28 | 0.14 | 0.14 | 2.80 | 6.11 | 2.35 | 2.22 | 5.12 | 8.11 | 7.00 | 5.94 | 6.47 |
| **6** | 0.11 | 0.10 | 0.09 | 0.10 | 9.98 | 5.06 | 6.88 | 2.03 | 9.29 | 11.78 | 12.80 | 11.40 |
| **7** | 0.47 | 0.26 | 0.46 | 0.31 | 2.99 | 5.59 | 3.47 | 4.24 | 12.91 | 15.66 | 13.64 | 17.79 |
| **8** | 0.11 | 0.24 | 0.23 | 0.24 | 0.93 | 3.33 | 1.17 | 1.24 | 13.05 | 14.26 | 6.20 | 8.90 |
| **9** | 0.60 | 0.10 | 0.15 | 0.25 | 5.16 | 0.66 | 1.70 | 0.57 | 10.13 | 3.35 | 4.50 | 5.15 |
| **10** | 0.07 | 0.06 | 0.08 | 0.58 | 0.65 | 0.99 | 0.33 | 1.16 | 2.35 | 3.91 | 1.01 | 4.74 |
| **11** | 0.19 | 0.13 | 0.30 | 0.13 | 4.94 | 1.24 | 4.84 | 2.37 | 10.00 | 5.94 | 6.48 | 7.22 |
| **12** | 0.52 | 0.09 | 0.20 | 0.08 | 3.24 | 1.39 | 2.05 | 1.08 | 6.63 | 3.07 | 6.90 | 5.73 |
| **13** | 0.39 | 0.25 | 0.25 | 0.44 | 2.00 | 2.41 | 4.43 | 4.44 | 5.24 | 7.55 | 13.18 | 11.57 |
| **14** | 0.30 | 0.42 | 0.08 | 0.22 | 3.96 | 6.93 | 7.58 | 4.48 | 10.33 | 15.02 | 16.43 | 6.17 |
| **15** | 0.20 | 1.05 | 0.38 | 1.09 | 1.50 | 4.65 | 1.62 | 6.94 | 11.85 | 12.84 | 16.50 | 11.68 |
| **16** | 0.47 | 0.64 | 0.33 | 0.45 | 2.22 | 1.22 | 2.49 | 0.54 | 17.85 | 23.15 | 6.58 | 5.19 |
| **17** | 0.17 | 0.11 | 0.27 | 0.22 | 0.81 | 0.34 | 2.68 | 0.98 | 3.13 | 2.72 | 4.34 | 4.70 |
| **Mean** | **0.30** | **0.30** | **0.25** | **0.49** | **2.98** | **3.26** | **2.83** | **3.00** | **8.79** | **10.50** | **8.65** | **9.05** |
| **S.E.** | **0.04** | **0.06** | **0.03** | **0.16** | **0.62** | **0.58** | **0.52** | **0.73** | **1.07** | **1.90** | **1.23** | **1.48** |
| **CS** |  | **0.9 rMT** | |  |  | **1.1 rMT** | |  |  | **1.3 rMT** | | |
|  | **Pre EL** | **Post**  **EL** | **Pre**  **LD** | **Post LD** | **Pre**  **EL** | **Post EL** | **Pre**  **LD** | **Post**  **LD** | **Pre**  **EL** | **Post EL** | **Pre**  **LD** | **Post**  **LD** |
| **1** | 0.74 | 0.49 | 1.43 | 0.11 | 1.38 | 0.90 | 1.37 | 0.83 | 4.81 | 5.43 | 6.48 | 4.39 |
| **2** | 0.09 | 0.29 | 0.36 | 0.53 | 4.60 | 2.23 | 1.75 | 1.64 | 3.91 | 4.70 | 6.16 | 2.73 |
| **3** | 4.90 | 0.38 | 0.22 | 0.18 | 3.95 | 3.62 | 4.23 | 1.10 | 6.21 | 5.13 | 6.03 | 6.44 |
| **4** | 0.31 | 0.19 | 1.06 | 0.23 | 9.22 | 0.72 | 3.60 | 2.84 | 15.84 | 3.25 | 9.50 | 6.20 |
| **6** | 0.38 | 0.60 | 0.37 | 0.51 | 1.42 | 1.96 | 1.66 | 2.58 | 7.24 | 8.81 | 11.07 | 12.02 |
| **7** | 0.37 | 0.97 | 0.55 | 0.47 | 26.60 | 24.04 | 2.79 | 4.02 | 28.73 | 23.78 | 3.81 | 8.41 |
| **8** | 0.53 | 0.66 | 0.46 | 0.58 | 5.95 | 7.29 | 5.20 | 6.46 | 10.25 | 9.91 | 8.95 | 8.78 |
| **9** | 0.17 | 0.15 | 0.18 | 0.19 | 5.46 | 2.74 | 2.59 | 3.99 | 7.60 | 3.75 | 6.71 | 6.94 |
| **Mean** | **0.94** | **0.46** | **0.58** | **0.35** | **7.32** | **5.44** | **2.90** | **2.93** | **10.57** | **8.09** | **7.34** | **6.99** |
| **S.E.** | **0.53** | **0.09** | **0.14** | **0.06** | **2.71** | **2.58** | **0.45** | **0.62** | **2.72** | **2.23** | **0.77** | **0.94** |

**Suppl. Table 1.** Ratios between motor evoked potentials and M responses (MEP/M, %) at intensities of 90% resting motor threshold (rMT), 110% rMT and 130% rMT in patients with migraine (MP) and control subjects (CS), pre and post standard room light exposure (EL) and pre and post light deprivation (LD). S.E. = standard error.
