# Supplementary material for: Increased variability of motor cortical excitability to transcranial magnetic stimulation in migraine: a new clue to an old enigma
Source: J Headache Pain. 2011 Sep 1;13(1):29–37. doi: 10.1007/s10194-011-0379-4 (PMC3253159; doi:10.1007/s10194-011-0379-4)
Supplement: Supplementary file 2 — Supplementary material 2 (DOC 77 kb) [file 10194_2011_379_MOESM2_ESM.doc]

|  | **Anxiety** | | | | | **Discomfort** | | | | |
| --- | --- | --- | --- | --- | --- | --- | --- | --- | --- | --- |
| **MP** | **Pre EL** | **Post EL** | **Pre LD** | **Post LD** | | **Pre EL** | **Post EL** | **Pre LD** | **Post LD** |  |
| **1** | 13.0 | 13.0 | 12.7 | 13.7 | | 24.0 | 26.4 | 24.4 | 24.9 |  |
| **2** | 14.2 | 3.9 | 9.2 | 10.7 | | 9.3 | 25.6 | 27.7 | 33.7 |  |
| **3** | 14.4 | 11.3 | 11.7 | 6.0 | | 14.2 | 29.8 | 29.3 | 24.6 |  |
| **4** | 12.8 | 6.9 | 12.3 | 11.7 | | 31.4 | 24.7 | 22.4 | 24.6 |  |
| **5** | 12.4 | 11.9 | 13.1 | 14.1 | | 27.9 | 27.6 | 24.0 | 30.3 |  |
| **7** | 10.0 | 9.5 | 12.3 | 9.5 | | 22.5 | 23.8 | 23.3 | 23.5 |  |
| **8** | 7.8 | 6.6 | 14.4 | 14.4 | | 28.0 | 26.1 | 27.0 | 27.4 |  |
| **9** | 13.0 | 12.1 | 12.5 | 12.9 | | 29.8 | 24.8 | 24.5 | 25.1 |  |
| **10** | 13.0 | 9.7 | 15.6 | 10.6 | | 31.1 | 26.0 | 28.7 | 26.4 |  |
| **11** | 17.5 | 7.3 | 14.2 | 13.5 | | 9.5 | 23.3 | 26.2 | 26.5 |  |
| **12** | 13.7 | 12.8 | 14.0 | 14.3 | | 35.3 | 24.6 | 26.8 | 25.1 |  |
| **13** | 10.0 | 12.1 | 7.5 | 8.1 | | 19.1 | 25.0 | 25.9 | 26.6 |  |
| **14** | 8.7 | 8.1 | 7.9 | 6.6 | | 23.4 | 20.3 | 18.0 | 11.5 |  |
| **15** | 14.4 | 11.9 | 9.7 | 11.6 | | 21.8 | 26.5 | 23.3 | 26.8 |  |
| **16** | 8.4 | 11.1 | 14.4 | 12.1 | | 9.2 | 24.7 | 25.3 | 27.1 |  |
| **17** | 14.5 | 7.9 | 8.1 | 9.2 | | 6.3 | 27.7 | 24.0 | 16.7 |  |
| **Mean** | **11.7** | **10.0** | **12.0** | **12.2** | | **20.2** | **25.5** | **25.2** | **25.2** |  |
| **S.E.** | **0.9** | **0.7** | **0.6** | **1.1** | | **2.4** | **0.5** | **0.6** | **1.2** |  |
|  |  | | | | |  | | | |  |
| **CS** | **Pre EL** | **Post EL** | **Pre LD** | | **Post LD** | **Pre EL** | **Post EL** | **Pre LD** | **Post LD** |  |
| **1** | 13.6 | 14.0 | 13.1 | | 14.1 | 23.7 | 24.6 | 23.3 | 25.8 |  |
| **2** | 11.1 | 12.2 | 11.3 | | 10.7 | 25.9 | 26.4 | 26.3 | 26.8 |  |
| **3** | 15.6 | 9.5 | 10.8 | | 19.9 | 27.2 | 27.9 | 28.1 | 26.0 |  |
| **4** | 8.1 | 7.9 | 9.0 | | 7.2 | 19.3 | 18.1 | 18.9 | 20.3 |  |
| **6** | 14.8 | 13.8 | 13.5 | | 12.7 | 30.6 | 30.3 | 31.2 | 27.8 |  |
| **7** | 13.4 | 12.7 | 8.1 | | 13.4 | 19.4 | 19.3 | 16.9 | 20.1 |  |
| **8** | 17.9 | 14.2 | 13.4 | | 14.6 | 27.1 | 27.3 | 27.2 | 26.5 |  |
| **9** | 14.7 | 13.6 | 12.2 | | 15.2 | 25.9 | 24.9 | 24.7 | 26.4 |  |
| **Mean** | **13.9** | **14.2** | **13.3** | | **14.5** | **25.0** | **25.4** | **24.6** | **25.3** |  |
| **S.E.** | **0.9** | **0.8** | **0.7** | | **1.1** | **1.2** | **1.4** | **1.5** | **1.0** |  |

**Suppl. Table 2.** Visual Analogue Mood State (VAMS) scores of anxiety and physical sedation in patients with migraine (MP) and control subjects (CS) pre and post standard room light exposure (EL) and pre and post light deprivation (LD) in transcranial magnetic stimulation experiments. S.E. = standard error.
