# Supplementary material for: Increased variability of motor cortical excitability to transcranial magnetic stimulation in migraine: a new clue to an old enigma
Source: J Headache Pain. 2011 Sep 1;13(1):29–37. doi: 10.1007/s10194-011-0379-4 (PMC3253159; doi:10.1007/s10194-011-0379-4)
Supplement: Supplementary file 3 — Supplementary material 3 (DOC 82 kb) [file 10194_2011_379_MOESM3_ESM.doc]

|  | **Sedation** | | | | **Cognitive impairment** | | | | |
| --- | --- | --- | --- | --- | --- | --- | --- | --- | --- |
| **MP** | **Pre EL** | **Post EL** | **Pre LD** | **Post LD** | **Pre EL** | **Post EL** | **Pre LD** | **Post LD** |  |
| **1** | 1.3 | 1.0 | 4.9 | 1.2 | 7.3 | 6.9 | 6.9 | 7.9 |  |
| **2** | 2.3 | 10.2 | 2.8 | 11.6 | 5.1 | 12.6 | 10.2 | 12.2 |  |
| **3** | 3.9 | 10.4 | 4.8 | 5.3 | 6.8 | 11.1 | 16.2 | 14.9 |  |
| **4** | 1.1 | 1.3 | 3.4 | 0.4 | 2.9 | 6.7 | 2.9 | 2.3 |  |
| **5** | 6.5 | 5.6 | 2.3 | 7.9 | 7.4 | 7.3 | 5.4 | 7.0 |  |
| **6** | 6.3 | 7.7 | 7.7 | 7.6 | 13.7 | 12.4 | 12.7 | 11.2 |  |
| **7** | 0.8 | 7.0 | 7.5 | 0.0 | 6.7 | 6.0 | 6.7 | 6.9 |  |
| **8** | 6.2 | 3.8 | 3.2 | 3.8 | 8.8 | 7.4 | 9.4 | 8.4 |  |
| **9** | 3.9 | 5.9 | 4.9 | 8.2 | 11.9 | 9.6 | 12.0 | 15.9 |  |
| **10** | 1.3 | 1.2 | 2.1 | 1.0 | 5.1 | 8.9 | 8.7 | 8.9 |  |
| **11** | 3.3 | 9.0 | 0.3 | 9.7 | 3.7 | 6.4 | 3.4 | 10.1 |  |
| **12** | 5.7 | 5.0 | 1.5 | 1.6 | 11.1 | 11.6 | 8.3 | 8.3 |  |
| **13** | 6.6 | 6.9 | 4.7 | 3.5 | 6.0 | 7.7 | 10.3 | 12.1 |  |
| **14** | 6.9 | 4.5 | 6.5 | 2.8 | 10.3 | 6.8 | 9.9 | 8.2 |  |
| **15** | 4.6 | 1.9 | 3.7 | 7.4 | 3.4 | 7.8 | 8.8 | 10.8 |  |
| **16** | 1.1 | 1.4 | 0.6 | 2.1 | 1.2 | 11.3 | 7.9 | 14.8 |  |
| **17** | 0.0 | 0.0 | 0.0 | 0.0 | 0.2 | 6.3 | 6.8 | 6.8 |  |
| **Mean** | **3.6** | **4.9** | **3.6** | **4.4** | **6.6** | **8.6** | **8.6** | **9.8** |  |
| **S.E.** | **0.6** | **0.8** | **0.6** | **0.9** | **0.9** | **0.5** | **0.8** | **0.8** |  |
|  |  | | | |  | | | |  |
| **CS** | **Pre EL** | **Post EL** | **Pre LD** | **Post LD** | **Pre EL** | **Post EL** | **Pre LD** | **Post LD** |  |
| **1** | 2.5 | 3.6 | 6.3 | 4.3 | 8.6 | 8.8 | 9.2 | 8.2 |  |
| **2** | 3.3 | 4.7 | 5.8 | 4.6 | 8.2 | 8.1 | 9.1 | 8.2 |  |
| **3** | 0.0 | 0.4 | 0.6 | 0.7 | 7.4 | 7.8 | 9.5 | 6.7 |  |
| **4** | 6.2 | 6.6 | 6.5 | 8.0 | 13.4 | 13.6 | 13.3 | 15.0 |  |
| **5** | 6.6 | 8.9 | 5.2 | 8.9 | 6.9 | 12.3 | 7.4 | 12.6 |  |
| **6** | 1.5 | 6.4 | 6.8 | 2.3 | 7.7 | 8.5 | 12.3 | 8.5 |  |
| **7** | 0.2 | 0.8 | 3.8 | 5.9 | 6.9 | 7.6 | 7.5 | 7.0 |  |
| **8** | 0.5 | 4.3 | 1.2 | 3.3 | 8.1 | 6.6 | 8.9 | 7.8 |  |
| **9** | 0.6 | 3.9 | 0.8 | 0.5 | 8.1 | 7.8 | 7.0 | 8.4 |  |
| **Mean** | **2.4** | **4.4** | **4.1** | **4.3** | **8.4** | **9.0** | **9.3** | **9.1** |  |
| **S.E.** | **0.8** | **0.9** | **0.9** | **1.0** | **0.7** | **0.8** | **0.7** | **0.9** |  |

**Suppl. Table 3.** Visual Analogue Mood State (VAMS) scores of mental sedation and other states in patients with migraine (MP) and control subjects (CS), pre and post standard room light exposure (EL) and pre and post light deprivation (LD) in transcranial magnetic stimulation experiments. S.E. = standard error of the mean.
